# Nothing About Us Without Us: Consolidating Experiences of Maternity Care to Advance Equity

**DOI:** 10.1111/1471-0528.18300

**Published:** 2025-07-24

**Authors:** Sarah G. Moxon, Wendy Graham, Veronique Filippi, Hannah Blencowe, Tanya Doherty, Tamar Kabakian‐Khasholian, Meghan Bruce Kumar, Dorothy Oluoch, Lisa Hinton

**Affiliations:** ^1^ Department of Infectious Disease Epidemiology and International Health London School of Hygiene and Tropical Medicine (LSHTM) London UK; ^2^ Health Systems Research Unit South African Medical Research Council (SAMRC) Cape Town South Africa; ^3^ Department of Paediatrics and Child Health University of Cape Town Cape Town South Africa; ^4^ Department of Health Promotion and Community Health, Faculty of Health Sciences American University of Beirut Beirut Lebanon; ^5^ Department of Nursing, Midwifery and Health Northumbria University Newcastle upon Tyne UK; ^6^ Health Services Unit KEMRI‐Wellcome Trust Research Programme Nairobi Kenya; ^7^ Nuffield Department of Primary Care Health Sciences University of Oxford Oxford UK

Progress in reducing levels of maternal and newborn mortality has stagnated in many settings since 2015—the start of the Sustainable Development Goals (SDGs), confirming enduring deep and complex structural and social inequities. This situation prevails despite both the increase globally in the proportion of women giving birth in health facilities and the shift in policy attention from coverage of interventions alone to effective coverage and the quality of facility‐based maternity care [1]. The persistence of poor experiences of maternity care in all settings is a particular concern, with some women and birthing people, due to their characteristics, facing greater vulnerability [2, 3], and now further amplified by the extreme and sudden cuts in global funding for MNH [4].

While efforts to improve the quality of maternity care are key to improving maternal and newborn health (MNH) outcomes, the focus on individual elements of quality (such as timeliness and evidence‐based practice) fails to sufficiently capture how this care is experienced by individuals. Despite growing recognition of experiences of care as a crucial determinant of overall healthcare quality, the concept of experiences of care remains poorly delineated, and thus difficult to measure and improve. A wide range of research on experiences of care is undertaken in different settings through different disciplines, applying different terms and methods to understand or describe similar phenomena [5, 6, 7, 8]. This commentary aims to highlight an important and timely imperative for those working to improve maternal and newborn health (MNH) outcomes, including practitioners, researchers and policymakers, to consolidate terminologies and methodological approches to studying experiences of maternity care (maternity care encompasses all care and services provided from onset of pregnancy to the end of the postnatal period both for women and their newborns, including experiences of bereavement care in the case of stillbirths, fetal loss or induced abortion). This effort is crucial to ensure that future research in this area is communicated clearly and effectively to key stakeholders. We argue that achieving more equitable maternal and newborn outcomes depends on translating complex care experiences into measurable improvements in policy and clinical practice.

Childbirth is a normal physiological process, albeit one with the potential for a wide range of short‐ and long‐term consequences for health and well‐being of both the mother and baby, through pregnancy and the postnatal period. Thus, experiences of maternity care differ from patient experiences more broadly. Precisely because pregnancy and childbirth are expected to unfold as a natural, positive event, there are often strong anticipations around how they should be experienced. Negative experiences of maternity care—whether clinical or interpersonal—can have lasting emotional and psychological impact on users and indeed providers.

Health professionals in maternity care services are both providers of clinical services and individuals with their own unique circumstances, vulnerabilities and who may also experience trauma and moral distress delivering care [9, 10, 11]. Many health providers in maternity and neonatal care suffer stress due to high workload, often due to insufficient human resources or lack of enabling environments to provide optimal quality of care [10, 11, 12, 13]. For those providing care in settings with high maternal and newborn mortality, relentless exposure to deaths without appropriate support mechanisms can lead to blame and fear, as well as emotional burnout [9]. Health provider experience of maternity care is not only important from a human rights perspective, but also directly influences how service users experience care, playing a key role in shaping experiences of maternity care overall [11].

The concept of inequity in healthcare has been valuable, but in many countries has tended to focus on differences in coverage, access to and utilisation of healthcare services, rather than inequities in experience of care, which can be shaped by intersectional social, cultural and structural systems that perpetuate injustices in maternal and newborn health [14]. These inequities manifest along a variety of dimensions, including gender norms, race, ethnicity, age, disability, religion, socio‐economic status and education, as well as structural factors driving vulnerabilities, such as conflict, forced displacement, migration and incarceration, meaning some women, due to their characteristics, are more likely to have poor experiences of maternity care [14, 15, 16]. Understanding experiences of maternity care within the context of these intersectional dimensions is essential to designing and implementing improvements in maternity services which respond effectively to individual needs [17].

The WHO's standards for improving maternal and newborn quality of care, published in 2016, placed experience of care as an equal tenet of quality, alongside the provision of care, thus pushing it into broader conversations on quality of care, especially from a perspective of equity, human rights and universal healthcare coverage [6]. The Lancet Global Health Commission on High Quality Health Systems called for better measures of health systems that are people‐centred, including people's confidence in the system and user experiences [8]. The Commission did not, however, specify which of the many different ways that user experience should or could be captured and shared. Experiences of health and illness, in this case experiences of maternity care and childbirth, can be recorded, analysed and (where necessary) improved by using a broad range of methods and types of data [18]. To support intersectional approaches, there is a growing body of evidence about how to meaningfully address ethnicity and race in health research. Deeper understanding of experience of care research methods, their implications, and how to communicate these experiences can be gained by learning from research conducted across all regions of the world [19, 20].

The wide spectrum of methods that can be used to understand experiences of care ranges from in‐depth narrative qualitative and ethnographic studies, focus groups, patient surveys and patient‐reported experience measures (PREMS) and participatory action research [5]. Not all these approaches generate equivalent types of data, and the deployment of each of these is often predicated on different purposes, which can range from situational analysis, national or regional service evaluation and small‐scale quality improvement. The heterogenous range of methodologies currently used to capture experiences of care presents challenges for evaluating and comparing interventions and policies [7, 21, 22]. There is a growing literature from a number of settings that describes data on patient experience, but a robust understanding of the different approaches, the evidence they provide, and how they can be used to improve care and translated into widespread implementation has not been rigorously established [23, 24, 25]. For example, considerable work has been undertaken in low‐ and middle‐income countries to develop standardised tools for measuring respectful maternity care [26, 27]. However, the different framings of experience of care from different contexts and disciplines have led to the use of overlapping concepts to describe related phenomena (such as respectful care, cultural competence, disrespect and abuse, mistreatment, obstetric violence) [28]. The multiple different approaches to measuring and evaluating experiences of care mean that research frequently operates in methodological and geographical silos, and opportunities for learning and knowledge sharing are not maximised, especially through sharing of lessons from the global south. In Box 1, we present three examples of experience of care research using different methods and approaches in diverse contexts in order to showcase opportunities for learning.

BOX 1 **Translating experience of maternity care research into equitable changes in policy, systems and practice: Examples from three settings.**

**Table:** 

Title	Aims	Context	Method(s)	How this research was used to implement change policy, systems, practice	Lessons learned
Exploring the potential for learning and using parent experiences of preterm birth to improve in‐patient neonatal care in Kenya	To incorporate outputs from mothers' narratives in developing communication training resources for multi‐professional newborn care teams	Nairobi, Kenya	Mixed methods using Experience Based Co‐Design (EBCD) approach: Video recorded narrative interviews with mothers Co‐design workshops Pre and post‐pilot training in‐depth interviews, and questionnaires	Participating nurses reported increased awareness of mothers' emotional needs and appreciation for empathy in care and respectful communication Institutional uptake with routine training of all the staff in the newborn unit The co‐designed training has been used to advocate for more structured, experience‐informed approaches to staff training The Nursing Council of Kenya has since accredited this training	Incorporating mothers' voices is essential for identifying gaps in care, and the insights gained can be used to design interventions that enhance the overall care experience
Maternal critical care: what can we learn from patient experience? [29]	To understand the experiences of women who experience a maternal near miss and require critical care after childbirth	Multiple sites, United Kingdom	Semi‐structured, narrative interviews with women in the UK who had experienced a maternal near‐miss Part of a wider programme of research on maternal morbidity [30] Published in peer‐reviewed paper [29] and on Health Experiences Insights (www.hexi.org)	Findings were used to inform guidelines published in 2018 by the Royal College of Anaesthetists in England to guide the care of a critically ill woman in childbirth [31] The research has informed clinical practice in England and Scotland and the development of networks of clinicians interested in maternal critical care research and practice, for example the Scottish Maternal Critical Care Network	Learning to inform policy and practice from in‐depth study of health experiences [29]
Strengthening teamwork and respect (STAR) in maternity units: developing a health system intervention in South Africa	To determine the impact of participatory learning and action groups among maternity health workers on teamwork and respectful care for women and newborns	KwaZulu‐Natal, South Africa	Mixed‐method approach: Development of an intervention toolkit and training programme [32] Quantitative surveys using person‐centered maternity care (PCMC) survey [33], and Dimensions of Learning Organisation Questionnaire (DLOQ) for maternity staff [34] Focus groups and in‐depth interviews with women, health professionals and managers	Findings highlighted differences between health professional cadres in learning opportunities and variation in women's experiences of care for themselves and their newborns Created and trained multi‐disciplinary teams in each maternity unit led by facility champions using a decide, plan, act, reflect and confirm cycle to improve teamwork and respectful care Priorities and gaps identified in the formative research guided the selection of participatory activities for the intervention PCMC survey highlighted communication issues, leading to activities that encourage health professionals to reflect on how women experience care Respectful care changes were implemented concerned with the care of women with stillborn babies and companionship in labour	Incorporating the voices of health professionals is crucial in designing interventions to improve experience of care

A further challenge is the fragmentation of language around approaches to bringing patient (or service user) voices into health service design, through approaches such as patient and public involvement and engagement (PPIE), community and/or participatory research and co‐design of services with communities and the users of health systems [35, 36]. All these approaches represent collective efforts to improve the impact of research by leveraging the voices of those with lived experiences of the phenomena under study and involve and engage them in decisions about research, including how data are interpreted, communicated and implemented for improvement [35]. To better understand and incorporate the voices of women, birthing people and service providers requires a better understanding of the nuances of different approaches and making appropriate adaptations to situations and contexts [35].

This commentary highlights the imperative for the research community to come together to share lessons and priorities to improve implementation research on experiences of maternity care. Table 1 begins the process of developing a prioritised research agenda. As suggested in the title ‘nothing about us without us’, the voices of women, birthing people, families and health providers are the driving source of knowledge and power in how to frame, understand and improve experiences of maternity care [28]. Yet a lack of clarity about the range of approaches to understanding experiences of care and incorporating service user and provider voices, the methods and types of data they can deploy and crucially, the types of evidence they can provide, is undermining and diluting their potential impact. Resolving this will require a diverse set of solutions and approaches from a variety of different disciplines and contexts, avoiding dichotomizing approaches from high and low‐income settings and recognising the potential for learning across geographical and economic boundaries. In this process it will be critical to maintain evolving feminist narratives and an anticolonial agenda in future language and framing to avoid diluting the structural factors that drive experiences of care, thus perpetuating the existing disparities. Moving this agenda forward will require creation of opportunities and mechanisms for knowledge exchange and capacity building, particularly on effective engagement with diverse stakeholders, including communities, policymakers, funders and health providers. Maternity care experiences are a universal phenomenon, but these experiences are not universally acknowledged. Understanding experiences of maternity care, the breadth of the concept and the different methods and languages used to study and describe experiences is essential to ensure these experiences play a role in how we implement policies and clinical care to achieve cultural competence. The goal of universal coverage of quality healthcare demands greater efforts from those working to improve maternal and health outcomes—clinicians, researchers and policy makers—to integrate the diverse and varied voices of individual experiences, and translate these effectively to implement impactful change.

**TABLE 1:** A proposed agenda for applied research on experiences of maternity care.

1.	Improve data collection, incorporating voice of women, birthing people and service providers in the design of data collection tools.
2.	Improve data analysis, incorporating voice of women, birthing people and service providers in the interpretation of the analysis.
3.	Work towards convergence of methods on experience of maternity care, where appropriate.
4.	Create understanding of the different concepts and language that are being used to understand experiences of maternity care and the implications of different language.
5.	Share wealth of existing data collection approaches to improve experiences of maternity care, especially from the global south.
6.	Create opportunities to share knowledge and improve understanding through research on the role of intersectionality in the experience of maternity care in certain groups, for example, migrants, ethnic minorities, single mothers, those living with disability and adolescents.
7.	Decolonise approaches to experience of maternity care research.

## Author Contributions

All authors contributed to the conceptualisation of the article. S.G.M. was responsible for writing drafting and writing of the paper with support from W.G., V.F. and L.H. All authors contributed sections of text and reviewed and edited multiple drafts.

## Conflicts of Interest

The authors declare no conflicts of interest.

## Data Availability

The authors have nothing to report.
